# Comparison of oral *Lactobacillus* and *Streptococcus mutans* between diabetic dialysis patients with non-diabetic dialysis patients and healthy people

**DOI:** 10.15171/jrip.2016.31

**Published:** 2016-07-09

**Authors:** Fahimeh Rezazadeh, Abdollah Bazargani, Jamshid Roozbeh-Shahroodi, Ali Pooladi, Peyman Arasteh, Khosro Zamani

**Affiliations:** ^1^Department of Oral and Maxillofacial Medicine, School of Dentistry, Shiraz University of Medical Sciences, Shiraz, Iran; ^2^Department of Bacteriology and Virology, Shiraz Medical School, Shiraz University of Medical Sciences, Shiraz, Iran; ^3^Shiraz Nephro-Urology Research Center, Shiraz University of Medical Sciences, Shiraz, Iran; ^4^Student Research Committee, School of Dentistry, Shiraz University of Medical Sciences, Shiraz, Iran; ^5^Non communicable Disease Research Center, Fasa University of Medical Sciences, Fasa, Iran; ^6^Student Research Committee, Shiraz University of Medical Sciences, Shiraz, Iran; ^7^Department of Bacteriology and Virology, Shiraz Medical School, Shiraz University of Medical Sciences, Shiraz, Iran

**Keywords:** *Lactobacillus*, *Streptococcus mutans*, Dental caries, Diabetes mellitus, Dialysis, Renal failure

## Abstract

**Introduction:** Diabetes is associated with higher rates of caries, on the other hand some studies have shown that renal failure can be protective against dental caries.

**Objectives:** In this study we compared oral Lactobacillus and Streptococcus mutans between diabetic dialysis and non-diabetic dialysis patients and the normal population.

**Patients and Methods:** During November 2014 to January 2014, 85 people that referred to our medical care center entered the study. The sample included 30 diabetic dialysis, 28 non-diabetic dialysis patients and 27 healthy people. Oral saliva samples were obtained from their tongue and oral floor for microbiological examination. Patients’ data were compared before and after dialysis.

**Results:** The amount of Lactobacillus and S. mutans did not show a significant difference between the three groups (P=0.092 and P=0.966 for S. mutans and lactobacillus, respectively). A positive and meaningful correlation was seen between fasting blood sugar (FBS) levels and the amount of S. mutans in the diabetic dialysis group (P=0.023; r=0.413). A meaningful and positive correlation was also seen between the amount of blood urea nitrogen (BUN) after dialysis and the amount of oral S. mutans in the non-diabetic dialysis group (P=0.03; r=0.403).

**Conclusion:** Despite the differences in the prevalence of caries that have been reported between renal failure patients and diabetic patients, we did not find any significant difference between diabetic dialysis, non-diabetic dialysis patients and the healthy population, regarding their amount of oral cariogenic bacteria.

**Implication for health policy/practice/research/medical education:**

Diabetic dialysis patients tend to have a complex oral status and special attention should be given to their oral health.

## Introduction

Diabetes is a common disease affecting more than 387 million people worldwide (1). The condition is associated with many complications and among them, diabetic nephropathy is one of the major complications that develops in 40% of patients with insulin dependent diabetes mellitus (DM) (2). The high prevalence of DM results in a sustained increase in the prevalence of diabetic nephropathy, which in return, causes increased kidney failure (3). Patients with DM are faced with more oral disorders than the normal population. Caries and periodontal disease are a well-documented complication of diabetes. Other complications include xerostomia, bacterial, viral and fungal infections and poor wound healing (4,5).

On the other hand, chronic renal failure is seen with several oral changes such as ammonia-like smell, dysgeusia, stomatitis, gingivitis, decreased salivary flow and parotitis (6). Although caries is not a characteristic of disease some oral lesions like lichen planus, hairy tongue and pyogenic granuloma have been reported with a higher rate in these patients (7).

The oral cavity is colonized with different species of bacteria and some of these bacteria contribute to some of the most common bacterial infections such as caries and periodontitis (8). The most important causes of dental caries are *Lactobacillus and Streptococcus mutans* (9).

Patients with diabetes are documented to have higher levels of dental caries due to multiple factors, on the other hand some studies have shown that patients with chronic renal failure may have lower levels of cariogenic bacteria in their oral cavity (4,10).

Different results regarding the oral microflora in patients with diabetes and renal failure have been reported (11-13). Ahmadieh et al (12) reported that hemodialysis and kidney transplantation could affect the oral microflora. They found that oral *Streptococcus mutans* and *Lactobacilli* were seen less frequently after kidney transplantation. In another study, the oral health of patients on hemodialysis for diabetic nephropathy and chronic glomerulonephritis was studied and they found that fewer teeth and a worse periodontal health was seen in diabetic nephropathy patients compared to other groups (4).

## Objectives

Due to the discrepancy in the prevalence of dental caries among these patients, in this study we compared oral *Lactobacillus and Streptococcus mutans* among patients with end-stage renal disease and diabetic nephropathy, both undergoing dialysis with the normal population.

## Patients and Methods

### Study settings and patients

This study was conducted in Faghihi hospital, Shiraz, Iran. The study population was selected from patients that were referred to our medical care center for dialysis, during November 2014 to January 2014. Patients who received periodontal treatment during the past year, brushed their teeth or ate something within one hour prior to sampling, took antibiotics or used mouth wash in the past previous month, were seropositive or suspicious to have HIV, hepatitis B or C, chronic smokers and patients that had any other systemic disease that could have affected the oral microflora were excluded from the study. They were then divided into two groups: group 1 or the diabetic dialysis patients, group 2 or the non-diabetic dialysis patients. A third group was selected from the healthy people who were accompanying the patients during the dialysis, as the control group.

### Sample collection

Oral saliva samples were collected from each patient using a swab. The swab was rotated to remove saliva from the dorsum of the tongue and the floor of the mouth. It was then immediately placed into a sterile bottle containing 1 mL of thioglycolate medium (HiMedia; India) in order to maintain the viability of the microorganisms collected. Within 15 minutes of collection, the swabs were transported to the microbiology laboratory for the microbiological testing.

For the enumeration of *Streptococcus mutans*, mitis salivarius agar (HiMedia, Indian) was used. The agar was supplemented with 0.1% potassium tellurite, bacitracin 0.2 U/mL, and sucrose 15% w/v (MSA). The samples were then diluted by 100 folds with salin, after which 10 µL of the diluted samples were transferred to the colony plates. The plates were then set in microaerophilic conditions and incubated for 1 day at 37°C.

For the *Lactobacillus*, Rogosa agar (HiMedia, India) was employed. For the enumeration of the bacteria, 20 µL of the samples was transported to the plates. The inoculated plates were then incubated in an anaerobic jar with an anaerobic atmosphere of 85% nitrogen, 5%-10% carbon dioxide and 5%-10% oxygen for 4 days at 37°C.

Assessment of the cultures and lab tests were all performed by a single microbiologist who was blinded to the study groups.

### Classification and evaluation

The salivary levels of the *S. mutans* were classified into five groups based on the colony count: 1: 0–10^5^ CFU/mL of saliva, 2: 10^5^-5×10^5^ CFU/mL, 3: 5×10^5^-10^6^ CFU/mL, 4: 10^6^ -1.5×10^6^ and 5: >1.5×10^6^.

The *Lactobacillus* were classified into six groups based on the colony count in each sample; 1: Non-detectable, 2: 1–10^3^ CFU/mL saliva, 3: 10^3^ –5×10^3^ CFU/mL, 4: 5×10^3^-10^4^ CFU/mL saliva, 5: 10^4^ -1.5×10^4^ CFU/mL and 6: >1.5×10^4^.

Patients’ information regarding age, sex, fasting blood sugar (FBS) levels, creatinine and blood urea nitrogen (BUN) levels prior to dialysis and after dialysis (only for the case groups) were recorded and compared between the groups.

### Ethical issues

The study protocol was in accordance with the declaration of Helsinki and was approved by the Ethics Committee of Shiraz University of Medical Sciences. All participants gave their informed written consent to enter the study (Ethical code# 2232).

### Statistical analysis

The statistical analysis was done using the SPSS^®^ for windows^®^, version 17 (SPSS Inc., Chicago, IL, USA). The data are displayed and compared as frequency, percent, median and interquartile range. The Kruskal-Wallis test was used to compare means among the 3 groups and the chi-square test was used for comparison of means between 2 groups. The Spearman’s correlation was used to assess the relationship between the frequency of each bacteria and different parameters including age, FBS, creatinine and BUN levels in the groups. A two-tailed *P* value of less than 0.05 was considered as statistically significant.

## Result

### Baseline characteristics

Eighty-five people entered the study and were evaluated. Group 1 (diabetic dialysis patients) included 30 patients, group 2 (non-diabetic dialysis patients) included 28 patients and group 3 (control group) included 27 healthy people. The patients did not display any significant difference regarding age and sex. The baseline characteristic of the participants is displayed in Table 1.

**Table 1:** Baseline characteristics and frequency of bacteria in the three groups^a^

**Variables**	**Group 1 (n=30)**	**Group 2 (n=28)**	**Group 3 (n=27)**	***P*** ** value**
Age (years)	65 ± 11	59 ± 14	63 ± 12	0.119
Sex (m : f)	20 : 10	17 : 11	17 : 10	0.893
FBS	194 ± 76	94 ± 19	–	<0.001
Creatinine before dialysis	6.5 ± 1.5	7.9 ± 2.3	–	0.008
Creatinine after dialysis	3.1 ± 0.8	3.5 ± 1.1	–	0.07
BUN before dialysis	54 ± 11	59 ± 17	–	0.26
BUN after dialysis	19 ± 5	19 ± 5	–	0.91
*Streptococcus mutans (*Frequency based on grouping^b^), n (%)				0.092
Group 1	2 (6.7)	4 (14.3)	1 (3.7)	
Group 2	7 (23.3)	10 (35.7)	7 (25.9)	
Group 3	7 (23.3)	5 (17.9)	1 (3.7)	
Group 4	3 (10)	1 (3.6)	4 (14.8)	
Group 5	11(36.7)	8 (28.6)	14 (51.9)	
*Lactobacillus (*Frequency based on grouping^c^), n (%)				0.966
Group 1	17 (56.7)	14 (50)	13 (48.1)	
Group 2	4 (13.3)	8 (28.6)	5 (18.5)	
Group 3	4 (13.3)	2 (7.1)	7 (25.9)	
Group 4	0 (0)	1 (3.6)	2 (7.4)	
Group 5	2 (6.7)	0 (0)	0 (0)	
Group 6	3 (10)	3 (10.7)	0 (0)	

^a^Group 1 includes the diabetic dialysis patients; group 2 includes the non-diabetic dialysis patients; group 3 includes the control group. ^b^ ****The *S. mutans* were divided into 5 groups based on their frequency in each group: group 1: 0-10^5^; group 2: 10^5^- 5×10^5^; group 3: 5×10^5^ – 10^6^; group 4: 10^6^ – 1.5 × 10^6^; group 5: > 1.5 × 10^6^.

### Comparison of groups and correlations

For comparison of the oral flora between the three groups, each of the two types of bacteria was classified into 5 groups as mentioned before. The results did not show a statistically significant difference in the amount of bacteria between the three groups (*P*=0.092 and *P*=0.966 for *S. mutans* and *lactobacillus*, respectively) (Table 1).

A statistically significant and positive correlation was seen between FBS and the amount of *S. mutans* in group 1, in other words an increase in the FBS of the diabetic dialysis patients resulted in an increase in the amount of oral *S. mutans* (*P*=0.023; r**=0.413), although this correlation was not found with *Lactobacillus* for this group (*P*=0.129; r**=0283).

In the non-diabetic dialysis group a meaningful and positive correlation was found between the level of BUN after dialysis and the amount of oral *S. mutans* (*P*=0.03; r**=0.403), although this correlation was not seen between BUN before dialysis and *S. mutans* for this group of patients (*P*=0.29; r**=0.205).

None of the other parameters showed a significant correlation with either of the bacteria (*P*>0.05; Table 2).

**Table 2:** Correlation coefficient between *Streptococcus mutans* and *Lactobacillus* frequency with differed variables in the three groups^a^

**Variables**	**Group 1 (n = 30)**				**Group 2 (n = 28)**				**Group 3 (n = 27)**			
	**SM (r)**	**P value**	**LB (r)**	**P value**	**SM.(r)**	**P value**	**LB (r)**	**P value**	**SM. (r)**	**P value**	**LB (r)**	**P value**
FBS	0.413	0.023	0.283	0.12	0.151	0.44	-0.234	0.23	-	-	-	-
Creatinine before dialysis	0.16	0.398	0.098	0.60	-0.66	0.74	-0.053	0.79	-	-	-	-
Creatinine after dialysis	-0.088	0.643	0.151	0.42	-0.64	0.74	0.152	0.44	-	-	-	-
BUN before dialysis	-0.025	0.894	0.22	0.24	0.205	0.29	-0.095	0.63	-	-	-	-
BUN after dialysis	-0.173	0.361	-0.25	0.17	0.403	0.03	0	0.99	-	-	-	-
Age	0.041	0.828	-0.258	0.168	0.396	0.037	-0.041	0.637	-0.054	0.78	-0.422	0.029

Abbreviations: FBS, fasting blood sugar; BUN, blood urea nitrogen; SM, *Streptococcus mutans*; LB, *Lactobacillus.* ^a^Group 1 includes the diabetic dialysis patients; group 2 includes the non-diabetic dialysis patients; group 3 includes the control group.

## Discussion

In our study we documented a positive correlation between the amount of FBS and the amount of oral *S. mutans* in the diabetic patients, furthermore the BUN after dialysis was positively correlated with the amount of oral *S. mutans* in the non-diabetic dialysis patients. No meaningful correlation between *Lactobacillus* and any of the disease related variables that we assessed in our study was seen.

In a recent study by Teratani et al (4) in 2013, the oral health was compared between patients with diabetic nephropathy (DN) and chronic glomerulonephritis (CGN) with a control group. They found that patients with diabetic nephropathy had overall worse oral health in compared to both CGN patients and the control group. Although both the CGN and the DN group both had a worse condition regarding salivary flow rate and total xerostomia score. The xerostomia score was worse in patients with DN compared to the CGN group. In another study in 2011 (14), comparing oral health among patients with DN and patients with other causes of chronic kidney disease (CKD) at pre-dialysis stage, they found that DN patients had higher rates of dental caries. Our study also showed a positive correlation between the amount of *S. mutans* and FBS in the diabetic patients, furthermore the rate of cariogenic bacteria did show a meaningful difference between the three groups. This could have been due to multiple reasons including that caries might not only be dependent on oral microflora and other factors such as salivary flow rates and the amount of sugar in the saliva could have affected the results, furthermore patients with renal failure tend to have higher pH of the oral cavity due to their uremic state which could have provided protection against dental caries (10).

In one study in 2010 (12), a higher rate of *S. mutans* was seen in the control group compared to patients with hemodialysis and renal transplant and a lower rate of lactobacilli was seen in the control group, although these differences were not significantly meaningful. This result was similar to our findings. This was further supported by other studies that found a lower rate of dental caries in end stage renal failure and renal transplant patients (15,16).

In one review by Soell et al in 2007 (17), they concluded that diabetic patients have an overall worse oral health in wise of dental caries when compared to healthy individuals. They concluded that this is due to two factors of increased oral sugar levels and increased *S. mutans* and by controlling these two factors oral caries becomes the same as in healthy individuals.

Similar to the previous study, one study in 2013 (18), evaluated the dental caries of diabetic (controlled and uncontrolled) and non-diabetic patients. They found that the diabetic group had the highest amount of oral *S. mutans* and lactobacilli in comparison to the other groups (*P*<0.001). They also found that the amount of caries in the uncontrolled diabetic group correlated positively with the amount of HbA1c (r**=0.574 and *P*=0.003), which is consistent with our findings regarding the correlations between the amount of *S. mutans* and FBS in our diabetic nephropathy group (r**=0.413 and *P*=0.023). Diabetic patients are documented to be at higher risks of developing conditions like periodontitis and xerostomia apart from the added nephropathy which acts as a separate risk factor for poor hygiene (19,20). As shown in the previously mentioned studies patients with diabetes have lower salivary flow rates, which put them at higher risks of caries. Similar result was also seen in other studies evaluating the cariogenic bacteria in diabetic patients (21).

The findings in our study suggest that the BUN after dialysis may reflect the oral health status, so further studies should be conducted to evaluate if BUN after dialysis could be used as an indicator of caries for non-diabetic dialysis patients. We also found that the higher blood sugar in diabetic patients reflects a worse oral health and this shows that the less the diabetes is controlled the more oral caries is expected in this group of patients. We found that the non-diabetic patients had a lower amount of cariogenic bacteria than the control group. In the diabetic dialysis patients, considering their diabetes, we expected a worse oral health as indicated by some of the previously mentioned studies (4,12,17), on the other hand considering their renal failure we expected less oral caries. In our study we found no difference in the amount of cariogenic bacteria in these patients compared to the other groups showing that these patients are complicated regarding their oral health status and special attention should be given to these patients’ dental health.

Larger scale studies considering different types of renal failure, patients with concomitant diabetes and in patients with different socio-economic status should be conducted to further compare the difference in the oral health of these patients.

## Conclusion

Despite the differences in the prevalence of caries that have been reported between renal failure patients and diabetic patients, we did not find any significant difference between diabetic dialysis and non-diabetic dialysis patients, regarding their amount of oral cariogenic bacteria. In renal failure patients the BUN level after dialysis was positively correlated with their amount of *S. mutans*, furthermore diabetic patients displayed more *S. mutans* by an increase in their FBS.

## Limitations of the study

Our study had some limitations. First the types of medications that the patients were using were not taken into consideration. This may have had adverse effects on their oral health and might have affected the results of our study. Another factor that might have affected the oral caries was the socio-economic status as indicated by one study (22). It was not taken into consideration as a factor in our study, although most of our recruited patients had a poor socio-economic status.

## Acknowledgments

The authors would like to thank the vice-chancellor of Shiraz University of Medical Sciences for their financial support of the study (grant# 7027). This study is based on the thesis for the D.M.D of Dr. Ali Pooladi. The authors would also like to thank Dr. Mehrdad Vosoogh from the Dental Research Development Center of the School of Dentistry for his contribution in the statistical analysis. The authors also like to thank Dr. Shahram Hamedani (DDS, MSC) for his editorial assistance in the manuscript.

## Authors’ contribution

All authors contributed to design of the research. AL, AB and KZ conducted the research. PA drafted the article. JR aided in the scientific revision of the manuscript. All authors read, revised and approved the final manuscript.

## Conflicts of interest

The authors declared no competing interests.

## Ethical considerations

Ethical issues (including plagiarism, data fabrication, double publication) have been completely observed by authors.

## Funding/Support

This is a thesis related to Shiraz University of Medical Sciences, School of Dentistry. (Institutional Review Board code #7027).
